# Autophagy and Mitophagy-Related Pathways at the Crossroads of Genetic Pathways Involved in Familial Sarcoidosis and Host-Pathogen Interactions Induced by Coronaviruses

**DOI:** 10.3390/cells10081995

**Published:** 2021-08-05

**Authors:** Yves Pacheco, Dominique Valeyre, Thomas El Jammal, Maxime Vallee, Fabien Chevalier, Jérôme Lamartine, Dominique Sigaudo-Roussel, Bernard Verrier, Dominique Israel-Biet, Nathalie Freymond, Vincent Cottin, Alain Calender

**Affiliations:** 1 Laboratory of Tissue Biology and Therapeutic Engineering, CNRS UMR5305, Claude Bernard University Lyon I, IBCP, 69007 Lyon, France; yves.pacheco@univ-lyon1.fr (Y.P.); thomas_3901@hotmail.fr (T.E.J.); fabien.chevalier@ibcp.fr (F.C.); jerome.lamartine@ibcp.fr (J.L.); dominique.sigaudo-roussel@univ-lyon1.fr (D.S.-R.); bernard.verrier@ibcp.fr (B.V.); 2 UMR INSERM 1272, University Sorbonne Paris Nord, Hospital Paris-Saint Joseph, 75014 Paris, France; dominique.valeyre@aphp.fr; 3 Department of Internal Medicine, Lyon University Hospital, 69007 Lyon, France; 4 Department of Bioinformatics, Hospices Civils de Lyon, 69007 Lyon, France; maxime.vallee@chu-lyon.fr; 5 Department of Pulmonology AP-HP, Georges Pompidou European Hospital, 75014 Paris, France; dominique.israel-biet@aphp.fr; 6 Department of Respiratory Medicine, Hospices Civils de Lyon, Lyon South Hospital, 69007 Lyon, France; nathalie.freymond@chu-lyon.fr; 7 Department of Respiratory Medicine, Hospices Civils de Lyon, National Reference Centre for Rare Pulmonary Diseases, Louis Pradel Hospital, Claude-Bernard Lyon 1, 69007 Lyon, France; vincent.cottin@chu-lyon.fr; 8 Department of Genetics, Hospices Civils de Lyon, University Claude Bernard Lyon 1, 69500 Bron, France

**Keywords:** sarcoidosis, COVID-19, SARS-CoV2, genetics, autophagy, TANK Binding Kinase 1, mitophagy

## Abstract

Sarcoidosis is a multisystem disease characterized by the development and accumulation of granulomas, the hallmark of an inflammatory process induced by environmental and/or infectious and or genetic factors. This auto-inflammatory disease mainly affects the lungs, the gateway to environmental aggressions and viral infections. We have shown previously that genetic predisposition to sarcoidosis occurring in familial cases is related to a large spectrum of pathogenic variants with, however, a clustering around mTOR (mammalian Target Of Rapamycin)-related pathways and autophagy regulation. The context of the COVID-19 pandemic led us to evaluate whether such genetic defects may increase the risk of a severe course of SARS-CoV2 infection in patients with sarcoidosis. We extended a whole exome screening to 13 families predisposed to sarcoidosis and crossed the genes sharing mutations with the list of genes involved in the SARS-CoV2 host-pathogen protein-protein interactome. A similar analysis protocol was applied to a series of 100 healthy individuals. Using ENRICH.R, a comprehensive gene set enrichment web server, we identified the functional pathways represented in the set of genes carrying deleterious mutations and confirmed the overrepresentation of autophagy- and mitophagy-related functions in familial cases of sarcoidosis. The same protocol was applied to the set of genes common to sarcoidosis and the SARS-CoV2-host interactome and found a significant enrichment of genes related to mitochondrial factors involved in autophagy, mitophagy, and RIG-I-like (Retinoic Acid Inducible Gene 1) Receptor antiviral response signaling. From these results, we discuss the hypothesis according to which sarcoidosis is a model for studying genetic abnormalities associated with host response to viral infections as a consequence of defects in autophagy and mitophagy processes.

## 1. Introduction

Auto-inflammatory diseases probably result from a defective interaction between the environment (whether through viral, bacterial, or microparticle antigens) and a genetically predisposed subject (possibly via an alteration of the innate immunity). Among these, sarcoidosis is a complex multifactorial granulomatous disease which implicates various etiological triggers, including environmental (mineral micro- and nanoparticles), infectious, and genetic factors [1]. The basic mechanisms leading to the development and diffusion of granulomas are currently unknown. Recent genetic studies performed by Genome Wide Association Studies (GWAS) or Whole Exome Sequencing (WES) in sporadic and familial cases of sarcoidosis suggested a synergistic role of various immune pathways. This includes a role for (1) specific human leucocyte antigen (HLA) class II haplotypes and innate immunity genes; (2) regulation of calcium ions and G-protein-coupled receptors; and (3) functional defects in autophagy regulation by intracellular metabolic platforms such as the mechanistic target of rapamycin (mTOR) complex and Ras-related C3 botulinum toxin substrate 1 (Rac1) hubs [2,3,4,5]. Sarcoidosis can occur in a hereditary context with a mode of transmission suggesting the presence of dominant mutations. Interestingly, the incidence rate of sarcoidosis varies from 15–20 cases per 100,000 in Northern European countries to less than 5–10 cases per 100,000 in South Europe, and a high incidence rate up to 30 per 100,000 in Afro Americans [1,6]. Geographic and/or ethnic variations in sarcoidosis incidence could be related to different genetic susceptibility to microbial (viral, bacterial, parasitic, or fungal) infections and/or region-specific mineral environmental exposures. The context of the pandemic (COVID-19) linked to the SARS-CoV2 (Severe Acute Respiratory Syndrome Coronavirus type 2) virus, which has killed more than 4 million people globally, reinforces the importance of the role of environmental gene interactions in the context of auto-inflammatory diseases [7]. Sarcoidosis mainly affects the lungs and SARS-CoV2, like SARS-CoV1 and the Middle East Respiratory Syndrome (MERS-CoV), can cause severe pneumonia and death in a significant proportion of people, which raises the question of the putative susceptibility of sarcoidosis patients to these highly infectious viruses [8]. Recent structural and functional host-pathogen interactome studies have identified around 332 cellular target proteins of SARS-CoV2 during the infectious process [9,10]. Among them, a substantial number of host proteins are involved in autophagy, an intracellular self-degrading process that is important for balancing energy sources at critical times during the development and in response to nutrient deprivation-induced stress. This observation raises the following questions: (1) at what level does the virus hijack host autophagy for replication; and (2) does the modulation of autophagy affect viral particle production and antiviral immunity?

Autophagy plays a housekeeping role in removing misfolded or aggregated proteins, clearing damaged organelles, such as mitochondria, endoplasmic reticulum, and peroxisomes, and eliminating intracellular pathogens [11]. Among a large spectrum of host perturbations, SARS-CoV2, like other coronaviruses, deregulates and hijacks autophagy [12]. The main goal of this work was to cross the list of mutated genes in familial forms of sarcoidosis and those published in Gordon and Hoffmann’s SARS-CoV2 host-cell interactome (SARS-CoV2-GHI) to identify a panel of common genes that we chose to call F-SARC-CoV. In 13 families predisposed to sarcoidosis, we observed 30 genes belonging to the SARS-CoV2 (GHI) host-cell interactome and, herein, discuss their function in the framework of pathways previously described in sarcoidosis pathogenesis. A control study was carried out in parallel with the same protocol on a series of 100 healthy individuals providing a list of 95 genes (Control-CoV panel) belonging to the SARS-CoV2 (GHI) host-cell interactome. The two panels of genes, F-SARC-CoV and Control-CoV, were then analyzed by the enrichment technique using the ENRICH.R software to identify the molecular pathways that could discriminate between the two groups. Our data suggested an imbalance of familial sarcoidosis genes in favor of autophagy- and mitophagy-related pathways, thus suggesting a putative abnormal response to viral infections in sarcoidosis patients.

## 2. Materials and Methods

### 2.1. Selection of Sarcoidosis Patients and Families

Sarcoidosis patients and families were selected from a national clinical network, the French Group on Sarcoidosis (GSF), which involves 28 French university departments of internal medicine and/or pulmonology. Clinical diagnostic criteria were assessed according to the American Thoracic Society/European Respiratory Society, the World Association of Sarcoidosis and other Granulomatous Disorders (WASOG) [13]. Sarcoidosis diagnosis was confirmed in all cases by histological analysis of either lymph nodes and/or bronchial biopsies. The familial forms of sarcoidosis were collected through the GSF-SARCFAM observational project, whose primary objective was to establish a clinical and genetic database on sporadic and familial sarcoidosis [14]. We selected 13 families (F1 to F13) with at least two or three first-degree relatives affected by sarcoidosis and including one or two healthy controls among siblings, five of whom (F1–F5) were previously published [4]. As a control analysis, we used exome data from a series of 100 healthy individuals belonging to the Department of Genetics in our hospital (University Hospital—Lyon, France). Genetic screening by whole-exome sequencing was approved by the ethical committee approval (CPP SUD EST-2-IRB 00009118—21 September 2016). The ClinicalTrials.gov- Protocol Registration and Results System (PRRS) Receipt under the reference NCT02829853 register of clinical and genetic studies. 

### 2.2. Genetic Analysis by Whole Exome Sequencing

Library preparation, exome capture, sequencing, and data analysis were performed with IntegraGen SA (Evry, France). Genomic DNA was captured using the Twist Human Core Exome Enrichment System (Twist Bioscience HQ, 681 Gateway Blvd South San Francisco, CA 94080, USA) and IntegraGen Custom. For a detailed explanation of the process, see the publication of Gnirke in *Nature Methods* [15]. Sequence capture, enrichment, and elution were performed according to the manufacturer’s instruction and protocols (Twist Bioscience) without modification, except for library preparation, which was performed with a NEBNext^®^ Ultra II kit New England Biolabs France, Genopole Campus 1, 91030 EVRY Cedex, France). For library preparation, 150 ng of each genomic DNA were fragmented by sonication and purified to yield fragments of 150–200 bp. Paired-end adaptor oligonucleotides from the NEB kit were ligated on repaired A-tailed fragments then purified and enriched by 7 polymerase chain reaction (PCR) cycles. A quantity of 500 ng of these purified libraries were then hybridized to the Twist oligo probe capture library for 16 h in a singleplex reaction. After hybridization, washing, and elution, the eluted fraction was PCR amplified (8 cycles), purified, and quantified by quantitative PCR to obtain a sufficient DNA template for downstream applications. Each eluted-enriched DNA sample was then sequenced on an Illumina NovaSeq as paired-end 100 reads. Image analysis and base calling was performed using Illumina Real Time Analysis with default parameters.

### 2.3. Bioinformatics

Sequence reads were mapped to the human genome build (hg38) using the Burrows–Wheeler Aligner (BWA) tool. The duplicate reads (i.e., paired-end reads in which the insert DNA molecules have identical start and end locations in the human genome) were removed. Variant calling for the identification of SNVs (Single Nucleotide Variations) and small insertions/deletions (up to 20 bp) was performed via the Broad Institute’s GATK Haplotype Caller GVCF tool (GATK 3.8.1) for constitutional DNA. Mutations with a QSS score below 20 and a VAF of tumor <0.02 were removed. Variants were processed through Ensembl’s Variant Effect Predictor (VEP, Version 95.1) for further annotation. This tool annotates variants and determines their effect on relevant transcripts and proteins, and predicts the functional consequences of variants. This tool is based on the data from the gnomAD database, in addition to the 1000 Genomes Project and KAVIAR databases. Moreover, an in-house database enabled sequencing artifacts to be filtered out. Five bioinformatics algorithms for pathogenicity networks were available to predict the functional, molecular, and phenotypic consequences of coding and non-coding SNPs. These included DANN, ALAMUT©, Mutation Taster, SIFT, and Polyphenv2 [16,17,18,19]. Other information, including quality score, homo- or heterozygote status, count of variant allele reads, and presence of the variant in the COSMIC database, were reported. Genotyping concordance between our patients and controls DNA and exome resulted in the identification of 96 SNPs. The ADVANTA™ (FLUIDIGM Corporate Headquarters, San Francisco, CA 94080, USA) Sample ID Genotyping Panel is a 96 SNP assay enabling laboratories to generate a sample-specific genetic fingerprint in order to confirm the identity between the results delivered and the received DNA sample. At the end of the bioinformatics process, the genotyping results were compared with the sequencing data and a concordance score was calculated. This enabled detection of failures in the sample preparation process (contamination, sample inversion, etc.) and ensured the concordance between the samples received and the results delivered. 

### 2.4. Selection of Pathogenic Gene Variants and Gene Set Enrichment Analysis

**Figure 1:**
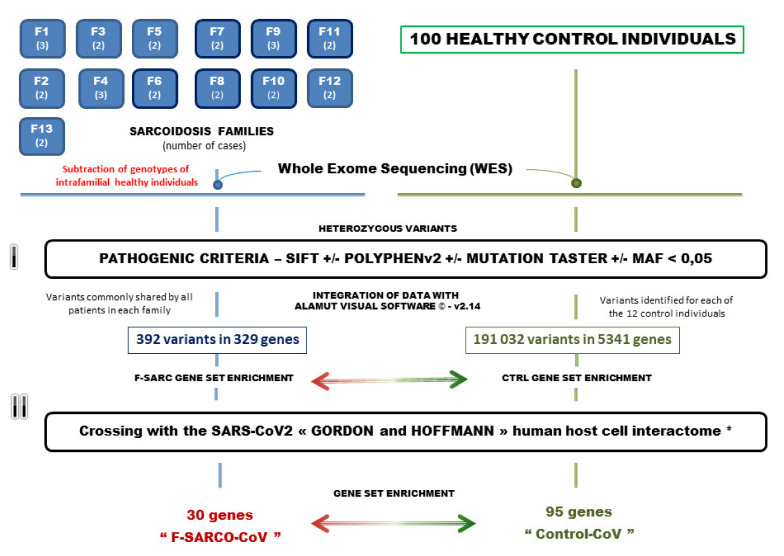
The procedure of gene variant selection in familial sarcoidosis and control individuals. Families F1 to F13 include 2 or 3 affected first-degree relatives (indicated in parentheses). Abbreviations: MAF, minor allele frequency. Alamut Visual^TM^ is an exploration software application for genomic variations from SOPHiA Genetics^TM^ (SOPHiA Genetics Inc., Interactive Biosoftware^TM^, Boston, MA 02116, USA).

Our study included 13 sarcoidosis families (F-SARC) that include 30 affected individuals. As previously reported, all sarcoidosis families collected through the SARCFAM project included two or more first-degree related patients and presented Mendelian features of autosomal dominant transmission [4,14]. For this reason, we selected only heterozygous gene variants present in all patients of a single family and absent from intrafamilial healthy individuals (one to two depending on the family). For the crossing of the genotypic data with the genes listed in the SARS-CoV2 (GHI) host-cell interactome, we analyzed in parallel a control series of 100 healthy individuals collected in our hospital (Lyon, France). As shown in Figure 1, the selection of gene variants used the following criteria: a Minor Allele Frequency (MAF) less than 0.05 (5%) and indication of in silico pathogenicity suggested by various software, such as SIFT and/or POLYPHENv2 and/or Mutation Taster and/or ALAMUT VISUAL^TM^ [16,17,18,19].

We only retained heterozygous variants (missense, nonsense, frameshift, in-frame deletions, and insertions) present in the coding regions (exons) of genes at or around the splicing donor (GT) and acceptor (AG) consensus sites. The genes sharing the selected variants were analyzed by ENRICH-R, a comprehensive updated resource and gene set enrichment system for a curated database, and a search engine that helps to identify clustered pathways and biological interactions of the proteins encoded by selected genes available at ENRICH.R website (Available online: https://maayanlab.cloud/Enrichr/, accessed on 24 May 2021) [20,21,22]. ENRICH-R computes three types of enrichment scores to assess the significance of overlap between the input list and the gene sets in each gene set library for ranking a term’s relevance to the input list: (1) the Fisher exact test, a test that is implemented in most gene list enrichment analysis programs; (2) a test statistic that we developed, which is the z-score of the deviation from the expected rank by the Fisher exact test; and (3) a combined score that multiplies the log of the *p*-value computed with the Fisher exact test by the z-score computed by the correction to the test.

### 2.5. Crossbreeding of the Genetic Data of Sarcoidosis and Control Individuals with the Genes Identified in the SARS-CoV2 Interactome

The list of mutated genes shared in both familial sarcoidosis patients and controls was crossed with the list of genes encoding target proteins of SARS-CoV2 in host’s cells (GHI) as recently established by expressing the tagged viral proteins in human cells, and allowing further identification of 332 host-viral protein-protein physical interactions (PPIs) by affinity purification mass spectrometry [9]. Using high resolution, CRISPR-Cas9 screening technology based on a single guide RNA (sgRNA) library targeting the 332 host proteins identified by the previous work, Hoffmann and coworkers confirmed the SARS-CoV2 intracellular PPIs and identified putative new relevant functional pathways related to viral protein targets in the host [10].

## 3. Results

### 3.1. Gene Variants Identified by the Selection Procedure

As described in Figure 1, the bioinformatics selection procedure was applied in a homogeneous manner to the patients from 13 sarcoidosis-predisposed families and the series of 100 unrelated healthy individuals. As previously published, we assumed that our study focused on genes acting as strong triggers in autosomal dominant forms of familial sarcoidosis, and as a consequence, we selected only heterozygous mutations shared by all (two or three) patients of a single family [4]. This explains the difference observed in the number of variants identified in the two series: 392 variants in 329 genes for familial sarcoidosis (F-SARC) and 191,032 variants in 5341 genes for the control series. An initial examination of the WES data indicates that some gene variants with a MAF higher than 0.05 may occur in more than one family. A MAF slightly greater than 0.05 (5%) was observed for 12 genes (TEP1, ADGRV1, SORBS3, TTC21A, DTHD1, ZAN, ATG9B, SACS, NLGN2, CCDC38, GLT8D2, and APPL2) in two families or more. Table S1 describes the whole F-SARC dataset. In a second step, we applied a MAF threshold at 0.05 in both familial sarcoidosis and control series. All the variants were then sorted using in silico pathogenicity criteria with SIFT, POLYPHENv2, Mutation TASTER, and ALAMUT VISUAL© (SOPHiA Genetics Inc., Interactive Biosoftware^TM^, Boston, MA 02116, USA) software [16,17,18,19]. As described in Table S1 for F-SARC, we identified 37 and 3 genes sharing mutations in two and three independent families, respectively. Interestingly, seven genes share the same variant in two independent families (PDE4DIP, rs111880480, F6/F8, MF = 0.01197—GOLGA2, rs41276654, F6/F8, MAF = 0.002821—GNAL, rs201898548, F6/F12, MAF = unknown—GPER1, rs138657567, F7/F11, MAF = 0.004058—PKD1, rs149151043, F7/F11, MAF = 0.006185—ZNF319, rs62637701, F7/F10, MAF = 0.001975—ANXA7, rs61732390, F8/F10, MAF = 0.00221).

### 3.2. Data Crossing with the SARS-CoV2 Gordon Hoffmann Interactome (GHI)

Among the 39 genes of the F-SARC cohort, 30 (named the F-SARC-CoV panel) also belonged to the SARS-CoV2 GHI [9,10] (Table 1). A similar crossing with the 5341 genes of the control series identified 95 members (named the Control-CoV panel) of the GHI (Table S2). Fifteen of the SARS-CoV2 target genes (highlighted in Table 1: AASS, ACAD9, ADAM9, BRD4, DNAJC11, FKBP10, FYCO1, IL17RA, NLRX1, PITRM1, PLAT, PLD3, PUSL1, TYSND1, and YIF1A) were present in both F-SARC and CTRL groups. The relative proportion of genes belonging to SARS-CoV2 GHI was, respectively, 9.1% (30/329) and 1.8% (95/5341) in the F-SARC and control series. However, given that the selection of variants in sarcoidosis families takes into account intrafamilial recurrence in patients, a statistical comparison of these percentages does not appear to us to be valid.

**Figure 2:**
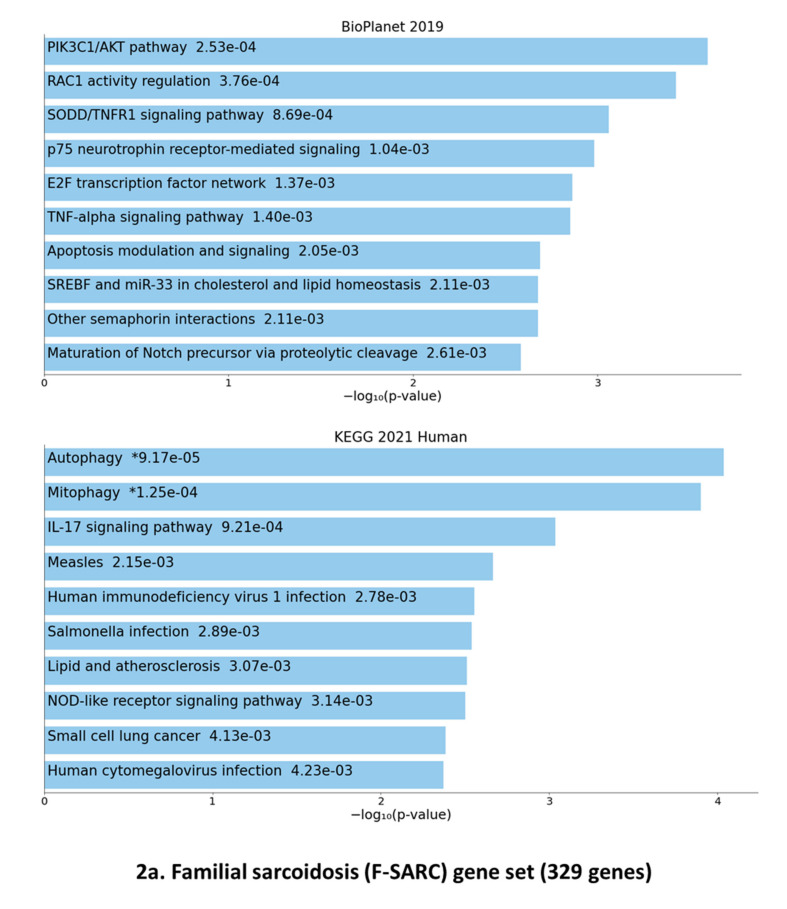

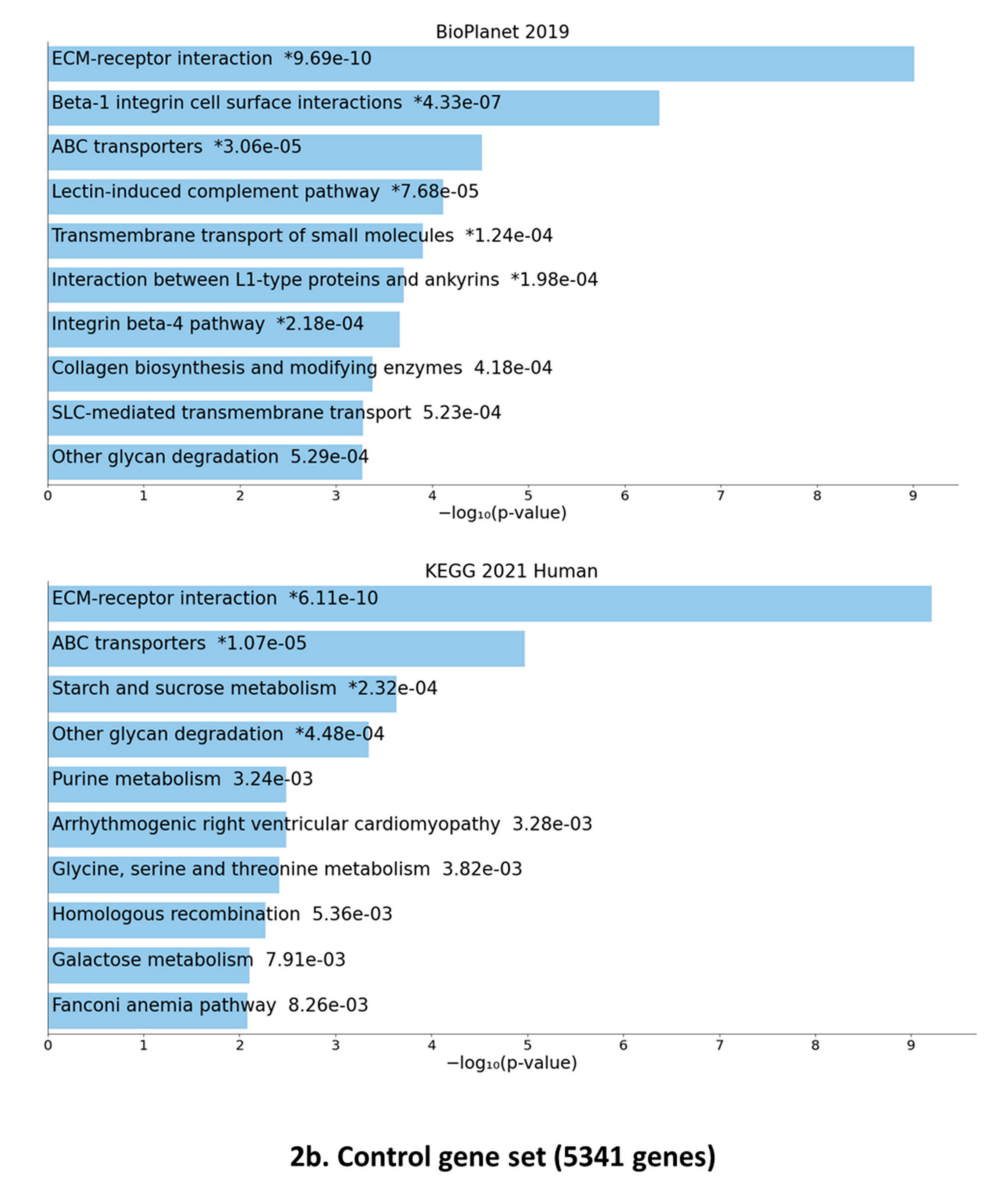
Disease-based enrichment analysis through the ENRICH.R web server [20,21,22]. Gene set enrichment analysis of the top 10 genes obtained through BIOPLANET-2019 (NIH-USA, National Center for Advancing Translational Science) and KEGG-2019 (Kyoto Encyclopedia of Genes and Genomes) pathway analysis on all of the deleterious variant carrying genes of F-SARC (**a**)—329 genes selected in familial sarcoidosis) and CTRL (**b**)—5341 genes selected in controls) series. The bar chart shows the top 10 enriched terms in the chosen library, with their corresponding *p*-values. Colored bars correspond to terms with significant *p*-values (<0.05). An asterisk (*) next to a *p*-value indicates the term also has a significant adjusted *p*-value (<0.05).

We first analyzed the full list of F-SARC and CTRL selected genes (329 for familial sarcoidosis and 5341 loci for control individuals) using the ENRICH.R gene set enrichment software (Available online: https://maayanlab.cloud/Enrichr/, accessed on 24 May 2021), which currently contains a large collection of diverse gene set libraries available for assessing pathways overrepresented by a specific set of candidate genes [20,21,22]. The resulting data were shown from two libraries: BIOPLANET-2019 (Available online: https://tripod.nih.gov/bioplanet/, accessed on 24 May 2021) and KEGG-2021 (Kyoto Encyclopedia of Genes and Genomes (Available online: https://www.genome.jp/kegg/pathway.html, accessed on 24 May 2021) (Figure 2a,b).

Statistical evaluation provided by the BIOPLANET database suggested a significant overrepresentation of PIK3C1/Akt (*p* = 0.000253) and Rac1 regulation (*p* = 0.000376) pathways for the F-SARC gene set (329 loci) followed by the SODD/TNFR1 signaling pathway (*p* = 0.000869). The KEGG human database enrichment highlighted three closely related intracellular pathways: autophagy (*p* = 0.0000917), mitophagy (*p* = 0.000125), and the interleukin-17 (IL-17) signaling pathway (*p* = 0.000921) (Figure 2a). A similar evaluation of the CONTROL gene set suggested a highly significant overrepresentation of different intracellular processes, such as extracellular matrix (ECM) receptor interaction (*p* = 0.000000000976—BIOPLANET and *p* = 0.00000000616—KEGG), Beta-1 integrin cell surface interactions (*p* = 0.000000437—BIOPLANET), ABC transporters (*p* = 0.0000307—BIOPLANET), and various other transmembrane pathways (Figure 2b). No significant overlap was found between the F-SARC and the CONTROL gene series.

**Figure 3:**
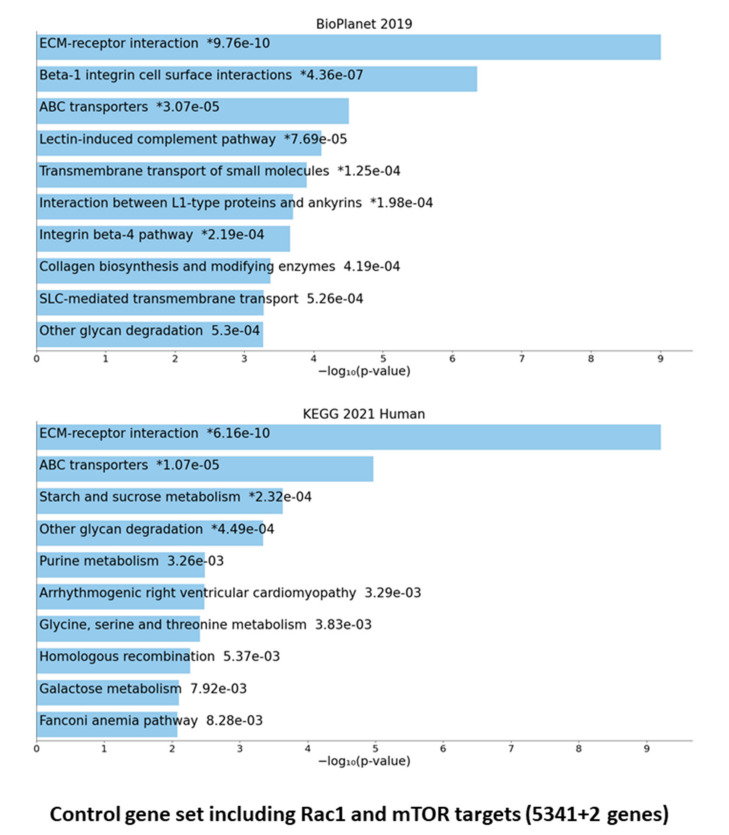
Gene set enrichment analysis of the top 10 genes obtained through BIOPLANET-2019 (NIH-USA, National Center for Advancing Translational Science) and KEGG-2021 (Kyoto Encyclopedia of Genes and Genomes) in the CONTROL gene set including the Rac1 and mTOR anchors. For this, the Rac1 and mTOR terms were included in the list of the CONTROL gene set inside ENRICH.R. The bar chart shows the top 10 enriched terms in the chosen library, with their corresponding *p*-values. Colored bars correspond to terms with significant *p*-values (<0.05). An asterisk (*) next to a *p*-value indicates the term also has a significant adjusted *p*-value (<0.05).

Our previous data on a subset of five sarcoidosis families suggested that genetic defects in Rac1, mTOR, and autophagy-related pathways are relevant factors in familial predisposition to sarcoidosis [4]. To evaluate a putative link between a significant number of control gene sets with Rac1 and mTOR, we artificially introduced the two Rac1 and mTOR gene symbols into the ENRICH.R software and analyzed the data with two databases of gene sets, BIOPLANET-2019 and KEGG-2021 (Figure 3). We did not observe any shift towards the Rac1 and/or mTOR pathway, and the top molecular pathways highlighted by the software were ECM receptor interaction (*p* = 0.000000000983—BIOPLANET and *p* = 0.00000000616—KEGG 2021).

The inclusion of Rac1 and mTOR in the F-SARC gene set emphasizes the clustering to PIK3C1/AKT- and Rac1-related pathways (*p* = 0.0000213 and *p* = 0.0000347, respectively, in BIOPLANET) and to autophagy/mitophagy (*p* = 0.00001392 and 0.0001302, respectively in KEGG 2021—BIOPLANET) and Rac1 activity regulation (*p* = 0.0000347—KEGG 2021) (data not shown). Despite the difference in the two gene set sizes (329—F-SARC and 5341—CONTROL), we estimate that our previous data linking the predisposition to inherited sarcoidosis to Rac1- and mTOR-related molecular hubs was confirmed in the 13 families analyzed in the present study.

### 3.3. Gene Set Enrichment of F-SARC-CoV and Control-CoV Gene Panels

**Figure 4:**
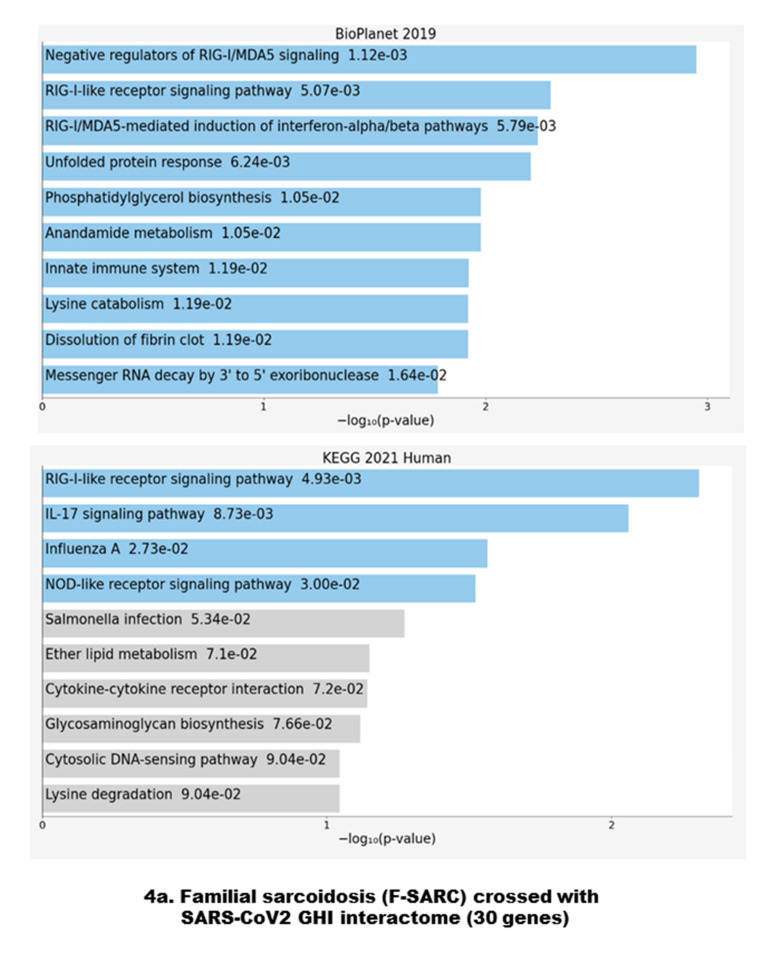

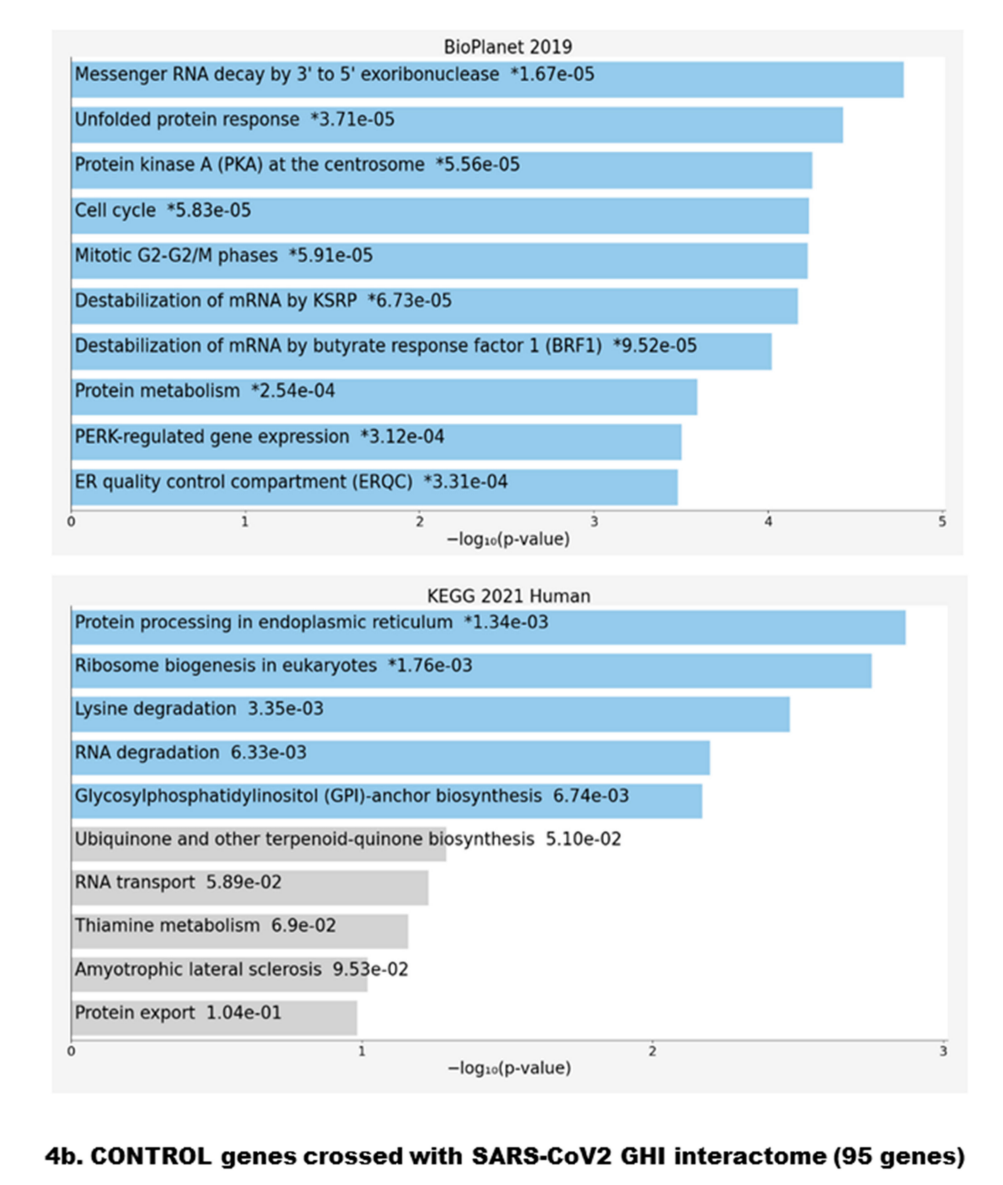
Disease-based enrichment analysis through the Enrichr web server [20,21,22]. Gene set enrichment analysis of the top 10 genes on the 30 F-SARC-CoV (**a**) and 95 CONTROL (**b**) genes sets crossing with the SARS-CoV2 GHI interactome. The bar chart shows the top 10 enriched terms in the chosen library, with their corresponding *p*-values. Colored bars correspond to terms with significant *p*-values (<0.05). An asterisk (*) next to a *p*-value indicates the term also has a significant adjusted *p*-value (<0.05). The data presented used two databases, BIOPLANET-2019 (NIH-USA, National Center for Advancing Translational Science) and KEGG-2021 (Kyoto Encyclopedia of Genes and Genomes).

As described previously, crossing the lists of genes sharing rare pathogenic variants in familial sarcoidosis and control series with the 332 genes included in the SARS-CoV2 host-cell interactome leads to two gene panels, F-SARC-CoV (30 genes) and CONTROL-CoV (95 genes), respectively. Figure 4 shows the functional network enrichment with BIOPLANET 2019 and KEGG 2021 through ENRICH.R. Genes mutated in familial sarcoidosis showed an overrepresentation of the RIG-1/MDA5 signaling pathway at three levels, negative regulation (*p* = 0.00112), steps involved in the activation of these cytosolic pattern recognition receptors (*p* = 0.00507), and downstream interferon-dependent inflammatory response (*p* = 0.00579). KEGG 2021 confirms these data (*p* = 0.00493) and implicates the IL-17 signaling pathway (*p* = 0.007873). Even if this overrepresentation was not associated with highly significant *p*-values, we observed a clear imbalance with those of the control group, focused on other molecular pathways, either non-sense mediated decay (NMD) and UPR with BIOPLANET 2019 (*p* = 0.0000167 and *p* = 0.0000371, respectively) or protein processing and ribosome biogenesis with KEGG 2021 (*p* = 0.00134 and *p* = 0.00176, respectively).

It seemed interesting to have a focus on the main features of the mutations observed in the 30 genes of the F-SARC CoV2 GHI (Table S3). All mutations identified have a MAF less than 0.05 (5%) and 20 of 30 genes shared very uncommon variants (MAF < 0.01 or unknown). The in silico evaluation of variants’ pathogenicity may be contradictory between different software (for example, SIFT vs. POLYPHENv2 vs. Mutation TASTER). In such a situation, the CADD (Combined Annotation-Dependent Depletion) is a good indicator of the pathogenic effect with a threshold established at 20. With the exception of PUSL1, all gene variants had a CADD near or greater than 20, suggesting they may be deleterious.

## 4. Discussion

At the crossroads of environmental, infectious, and genetic factors, sarcoidosis is a complex multifactorial disease belonging to the large spectrum of auto-inflammatory diseases. A major question has been raised about the susceptibility of patients to COVID-19, which affects predominantly, but not only, the respiratory system and the digestive tract, and the central nervous and vascular systems [23]. This point of vigilance is even more critical in patients sharing germline mutations in genes involved in innate immunity and/or key intracellular processes such as autophagy, as suggested in Crohn’s disease, sarcoidosis, and other chronic granulomatous diseases. [24,25]. Several case reports and a large population cohort analysis have been recently published and considered that experiencing chronic respiratory lung disease may result in increased morbidity and mortality putatively linked to the pre-existing parenchymal lesions and/or the impact of immunosuppressive therapies [26]. Sarcoidosis patients appear to have a modestly increased risk (OR ≈ 1.5) of severe COVID-19 putatively related to the previous lung function impairment [27].

Our study is the first to cross the genes involved in an auto-inflammatory disease and the host-cell interactome of SARS-Cov2, thus highlighting putatively common pathways involved in both sarcoidosis pathogenesis and viral infection. In autosomal dominant forms of sarcoidosis and based on the SARS-CoV2 GHI, the results of our study confirmed a significant accumulation of pathogenic variants in genes regulating the RIG-I (Retinoic acid-Inducible Gene I)–MAVS (Mitochondrial Antiviral Signaling Protein)-dependent type I IFN (Interferon) signaling pathway (Figure 4a). In contrast, genes of the control series crossing with the SARS-CoV2 interactome focus significantly various intracellular pathways such as regulation of messenger RNA decay, unfolded protein response (UPR), protein processing in endoplasmic reticulum, cell cycle and centrosome-related and mitotic regulation (Figure 4b).Even if the number of genes crossing with GHI differs between the F-SARC series (30 genes) and controls (95 genes), these results indicate an imbalance of the intracellular targets of the viral proteins. This observation suggests that the genes involved in the familial forms of sarcoidosis reveal one of the major mechanisms of the deleterious effects of infection with the SARS-CoV2 virus, in agreement with the data published in severe forms of COVID-19 [28].

### 4.1. Antiviral Immune Signaling and Autophagy

As suggested in previous studies, a large number of immunological and biochemical data suggest that deficiency in the clearance of bacterial and/or viral and/or inorganic particles related to autophagy defects and concomitant various immune defects—including the occurrence of significant Th1/Th17 immune responses initiated by antigen presentation and impaired regulatory T cell functions—may be associated with human sarcoidosis [1,5,25]. The cell entry of SARS-CoV2 depends on the binding of the viral spike (S) proteins to the angiotensin converting enzyme 2 (ACE2) receptor, expressed on mucosal and bronchial cells in humans, concomitantly with the serine protease transmembrane protease serine 2, which mediates S protein cleavage (S1 + S2) and maturation [28]. This molecular interaction leads to a membrane fusion process and promotes the formation of syncytia with multinucleated alveolar epithelial cells and inflammatory clusters [29]. Molecular and in silico studies have shown that the viral S protein binds to GRP78 (glucose-regulated protein 78) and heat shock proteins’ cell surface receptors known to activate autophagy through the AMP-kinase mTOR pathway [30]. SARS-CoV-2 contains a positive-sense single-stranded 30 kb RNA genome gathering fourteen open reading frames (ORFs) underlying various conserved genes (ORF1ab, S, E, M, and N protein region). Among various SARS-CoV2 proteins, spike (S), envelope (E), membrane (M), and nucleocapsid (N) are the four structural proteins contributing to the assembly and release of new viral particles within infected cells in humans [31]. As shown by Gordon, Hoffmann and coworkers, the virus affects several intracellular functions through a minimum of 332 host protein interactions. Some of these interactions are linked to the host reactivity and response, including the activation of autophagy, the induction of apoptosis, and the activation of the endoplasmic reticulum stress pathway (ER-STRESS) through the induction of the UPR (unfolded protein response) process [9,10,32,33,34]. The host defense will be activated, with the single strand viral RNA acting as a pathogen-associated molecular pattern (PAMP) that triggers the production of type I (IFN-α/β) and type III interferons [35]. Once the virus has entered the cell by endocytosis, the viral genomic RNA is translated directly into two poly-cistronic frames in a pp1ab protein whose cleavage will generate the series of NSP, from 15 to 16 depending on the strains. One of these NSPs (12) encodes an RNA-dependent RNA polymerase, allowing the production of a transient double stranded viral RNA (dsRNA), which is detected by TLR3 in endosomes and MDA5 in the cytosol [36,37]. Upon such PAMP recognition, two major pathways are activated: (1) the NF-ƙB pathway through the TRIF (TIR-domain containing adapter inducing IFN)–RIP1 (Receptor Interacting Protein 1)–TRAF6 (TNF-receptor-associated factor 6) and TBK1 (Tank Binding Kinase 1) molecular cascade; and (2) the induction of genes encoding type I IFNs and other proinflammatory cytokines through the direct phosphorylation of IRF3 by TBK1 [38].

**Figure 5:**
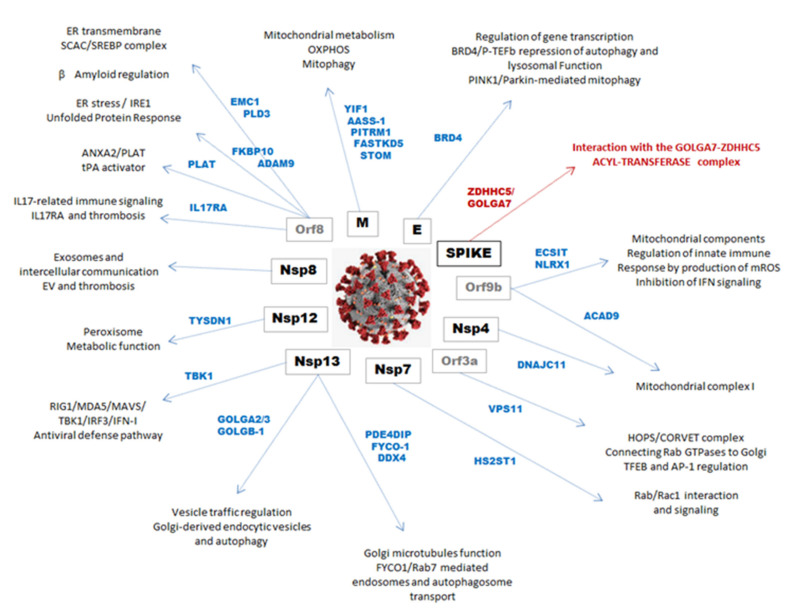
Schematic representation of the interactions of the proteins of the SARS-CoV2 virus with the proteins encoded by the mutated genes in the familial forms of sarcoidosis, with a summary of the intracellular functions concerned. Abbreviations: Nsp, non-structural viral proteins; Orf, open reading frame. The gene acronym descriptions are in the text.

This brief review of the mechanisms involved in COVID-19 pathophysiology allows us to hypothesize about the putative role of the familial sarcoidosis-related genes (F-SARC-CoV2) in the framework of the antiviral response induced by SARS-CoV2 infection. Figure 5 provides a schematic representation of protein–protein interaction (PPI) of the F-SARC-CoV gene panel.

The most represented viral proteins in the F-SARC-CoV2 interactome are M (membrane protein) and NSP13. M is the most abundant protein produced by coronaviruses and has the ability to neutralize antibodies produced in SARS-CoV2 infected patients [39]. M interacts with the central adaptor protein MAVS, which drives antiviral innate immunity in response to RNA virus infection and, as a consequence, inhibits the production of type I/III IFNs induced by the cytosolic dsRNA-sensing pathways mediated by RIG-I/MDA-5-MAVS [40]. Impaired MAVS aggregation may affect its recruitment to the downstream signaling pathways involving TRAF3 (TNF-Receptor Associated Factor 3), TBK1 (Tank Binding Kinase 1), and IRF3 (Interferon Regulatory factor 3), and a putative decrease in the innate antiviral response. Interestingly, MAVS may be phosphorylated by TBK1 in response to stimulation, and thereby binds to and recruits interferon regulatory factor 3 (IRF3) for its phosphorylation and activation by TBK1 [41]. TBK1, which is a target of NSP13, also plays a critical role in autophagy by regulating the mTOR complex. Activation of the metabolic checkpoint kinase mTORC1—A major component of the mTOR complex (mTORC1)—Has been shown to induce excessive granuloma formation in a knockout murine model by conditional inactivation of *TSC2* in monocytes (Tuberous Sclerosis Complex 2), an upstream inhibitor of mTOR [42]. Dysfunction of mTOR in sarcoidosis has been highlighted by genetic screening of familial forms of the disease [4,25]. We may expect that the TBK1 mutation observed in our study (F1 family) disturbs the role (both by activation or inhibition) and/or the function of this gene regarding both interferon production and autophagy regulation. TBK1 plays this role by modulating mTOR, and it is well documented that both DNA (e.g., herpes viruses) and RNA viruses, such as all strains of coronavirus, modulate the mTOR pathway by activation of the PI3K/Akt pathway and/or mTOR itself [43]. Another impact of this mutation may be a dysregulation of mitophagy, or the degradation of dysfunctional or damaged mitochondria. Specifically, Rab7A GTPase phosphorylation by TBK1 promotes mitophagy via the PINK/PARKIN ubiquitin ligase pathway [44]. TBK1 phosphorylates various autophagy modifiers and receptors during various steps of autophagosome formation. Interestingly, recent studies on host genetic susceptibility to severe forms of COVID-19 have shown that germline mutations of TBK1 could be a predisposing factor to SARS-CoV2-induced acute respiratory distress syndrome and severe and/or fatal prognosis of infected patients [45]. Functional studies will be necessary to assess the real impact of mutations in the TBK1 gene, both on autophagy and on interferon production. Numerous clinical observations have shown the occurrence of sarcoid-like granulomas after the institution of recombinant type I interferon therapy in various clinical conditions such as viral hepatitis [46,47]. In acute sarcoidosis, CD4 T lymphocytes produce large quantities of various cytokines and interferon gamma. Based on these data, we must consider that the impact of genetic defects in TBK1-related pathways is probably very important in terms of its role in mitophagy, a process well known to be an important regulatory mechanism controlling inflammatory responses in immune cells.

Close to autophagy regulation, an interesting gene of the F-SARC-CoV2 interactome is BRD4 (Bromodomain-containing protein 4), which has been shown to interact with E, the envelope protein of SARS-CoV2 acting as an ion channel and allowing host-cell proteases to release the viral genomic material [48]. BRD4 is a member of the Bromodomain and extra-terminal (BET) family proteins, which act as genetic readers of histone acetyl-lysine residues to regulate gene transcription [49]. BRD4 interacts with the positive-elongation factor (*p*-TEFb) complex, a critical factor for transcription elongation that acts as a transcriptional repressor of autophagy and lysosomal function [50]. Recent studies in a mouse cardiovascular model showed that upregulation of BRD4 inhibits PINK1/Parkin-mediated mitophagy, resulting in the accumulation of damaged mitochondria [51]. The mutation observed in one of the sarcoidosis family is a missense (p.Ser494Leu) with a high pathogenicity score (CADD = 28.9) and very low frequency (MAF = 0.000009951) affecting the N-terminal cluster of phosphorylation sites (NPS) and breaking the link with chromatin-linked transcription factors such as p53 [52].

### 4.2. Mitochondrial Dysfunction

We showed that mitophagy and the mitochondrial complex I assembly model OXPHOS system were overrepresented in the sarcoidosis-mutated genes and the SARCO-CoV2 interactome, respectively (Figure 2 and Table 1). For example, PITRM1 (pitrilysin metallopeptidase 1) (PITRM1), a mitochondrial matrix enzyme, digests oligopeptides, including the mitochondrial targeting sequences that are cleaved from proteins imported across the inner mitochondrial membrane [53]. PITRM1 deficiency leads to mitochondrial unfolded protein response and enhanced clearance, a defect associated with various neurodegenerative diseases underlying a novel mitochondrial-related neurodegenerative phenotype associated with β-amyloid protein accumulation [54]. One may expect that SARS-CoV2 M protein and PITRM1 interaction will contribute to a decrease in mitochondrial function in SARS-CoV2-infected cells. In sarcoidosis, the impact of mitochondrial dysfunction has been poorly explored but more extensively studied in lung fibrosis, a hallmark of severe forms of sarcoidosis [55,56,57]. M2 macrophage polarization is related to the initial steps of granuloma formation and it is well known that pathogens can affect mitochondrial metabolism and shape the macrophage response of proinflammatory (M1) versus immunomodulatory (M2) polarization [58,59,60]. Inhibition of mitochondrial oxidative phosphorylation is one of the main factors responsible for preventing macrophage M1 to M2 repolarization, which might be an underlying mechanism for worsening the disease [61].

Interestingly, three genes from the F-SARC-CoV2 interactome (YIF1A, FASTKD5, and AASS) also have significant regulatory roles in mitochondria metabolism. YIF1A interacts with a complex belonging to the ER, vesicle-associated membrane protein-associated protein B (VAPB), and the mitochondrial membrane protein, protein tyrosine phosphatase interacting protein-51 (PTPIP51), involved in signaling between the ER and mitochondria, an interaction whose dysfunction has been related to neurodegenerative diseases [62]. The VAPB-PTPIP51 complex regulates autophagy by modulating calcium delivery at ER-mitochondria contact sites [63]. These data highlight the functional importance of calcium homeostasis in sarcoidosis pathogenesis. AASS-1 encodes a bifunctional enzyme (α-aminoadipic semi aldehyde synthase) whose catalytic function elevates the lysine catabolic intermediate saccharopine, a product that disrupts mitochondrial dynamics and induces organelle damage [64]. FASTKD5 (Fas-activated serine-threonine kinase family protein-5), a member of the FASTK RNA binding proteins and a bona fide component of mitochondrial RNA granules (MRGs), associates with NLRX1 (NOD-like receptor member family X1), encoded by the NLRX1 gene that we found to share mutations in both F-SARC and control series (Table 1) [65]. This complex regulates the abundance of probably all mitochondrial genomes and the transcription of key components such as the respiratory complexes I and IV [66]. Taken together, these data suggest that the SARS-CoV2 M protein has a central role in mitochondrial dysfunction during viral infection, and our observation raises the question of the potential consequences of mutations of these target genes in patients with sarcoidosis.

Regarding TBK1, three genes designed as host targets of SARS-CoV2 *ORF9b*—*ECSIT* (Evolutionarily conserved signaling intermediate in Toll pathway), NLRX1 (nucleotide-binding oligomerization domain, leucine rich repeat containing X1), and *DNAJC11* (DnaJ Heat Shock Protein Family (Hsp40) Member C11)—Are mitochondrial components involved in the regulation of the antiviral innate immune response. ECSIT is an adapter protein of the Toll-like and IL-1 receptor signaling pathways involved in the activation of NF-kappa-B via MAP3K1 [67]. NLRX1 has been shown to negatively regulate type-I interferon, attenuate pro-inflammatory NF-κB signaling, promote reactive oxygen species production, and modulate autophagy [68]. DNAJC11 has been involved in mitochondria homeostasis by stabilizing membrane curvature and forming contact sites between the mitochondrial inner and outer membranes [69]. All of these functions have been related to the functional effect of ORF9b, which inhibits RIG-1-MAVS antiviral signaling during the host’s cell infection, and manipulates the host’s cell mitochondria and mitochondrial function to help SARS-CoV2 escape the host’s innate immunity, as shown with previous strains of coronaviruses (e.g., SARS-CoV1) [10,70]. This pathway was most represented in both F-SARC-CoV2 genes as putative crucial targets of SARS-CoV2 during COVID-19 (Figure 4 and Figure 5).

### 4.3. Rho GTPases Related Pathways

The viral non-structural NSP13 protein interacts with six proteins encoded by genes (GOLGA2, GOLGA3, GOLGB1, TBK1, FYCO1, and PDE4DIP) sharing mutations in familial sarcoidosis (Table S1). NSP13 belongs to the NSP1-16 replicase-transcriptase complex (RTC) of SARS-CoV2 and encodes a helicase/triphosphatase [71]. Golgins regulate the Golgi architecture, cell polarity and migration, membrane trafficking, endocytic vesicles and fusion with lysosomes, and, finally, autophagy in close interaction with various Rho GTPases such as Rab, Rac1/Cdc42, and RhoA [72]. A closely related function has been shown for FYCO1 (FYVE And Coiled-Coil Domain Autophagy Adaptor 1), a multidomain adaptor protein that plays an important role in autophagy by mediating the kinesin-dependent microtubule plus-end-directed transport of autophagosomes [73]. The functional role of FYCO1 in autophagy implicates its binding to Phosphatidylinositol-3-phosphate (PI3P), a crucial lipid in membrane dynamics, Rab7 GTPase, and LC3 (Microtubule-associated protein 1A/1B-light chain 3). FYCO1 is further required for the association of autophagosomes and lysosomes with the ribonucleoprotein (RNP) granules, which play a major role in compartmentalizing cytoplasmic RNA [74]. FYCO1 has molecular interactions with small GTPases from the Ras superfamily and is a candidate gene in a 3p21.31 gene cluster significantly associated with susceptibility to acute respiratory failures during COVID-19 [75]. Finally, PDE4DIP (phosphodiesterase 4D interacting protein or myomegalin), whose gene was found to be frequently mutated in familial cases of sarcoidosis, interacts with SARS-CoV2 protein NSP13 and should be considered due to its close interaction with phosphodiesterase 4D, which is anchored at the centrosome-Golgi region and plays a functional role in microtubule formation [76].

As discussed in a previous work, the Rac1-mTOR-autophagy axis appears to be a critical pathway involved in genetic predisposition to sarcoidosis [4,5,42]. Mechanistically, Rac1 binds mTOR and regulates mTORC1 cellular localization and activity. Any factors interacting in this binding may regulate autophagy. In the immune context, pro-inflammatory and anti-inflammatory cytokines can regulate (activation or inhibition) autophagy by acting on this interaction. A major example is TNFAIP8L2/TIPE2 (tumor necrosis factor, alpha-induced protein 8-like 2), a newly identified immune negative regulator of innate and adaptive immunity, which can compete with mTOR for binding the GTP-bound activated state of Rac1 and negatively regulate mTOR activity [77]. Another important cellular factor is Transcription factor EB (TFEB), a master regulator of autophagy and lysosome biogenesis. Rac1 GTPase is a negative modulator of autophagy by targeting TFEB [78]. Mechanistically, Rac1 reduces autophagy flux by repressing the expression of autophagy genes and mTOR phosphorylates TFEB to facilitate the interaction between GTP-bound forms of Rac1 and TFEB. This inhibitory interaction prevents the dephosphorylation and nucleus translocation of TFEB, which hampers the transcriptional activation of autophagy-related genes. The functional effect of Rac1 on MTOR is strongly regulated by various tissue-specific GEF (Guanine Exchange nucleotide Factors) such as Rho guanine nucleotide exchange factor 25 (ARHGEF25) [79].

### 4.4. Interleukin-17-Related Pathways

SARS-CoV2 *ORF8* host targets belonging to the sarcoidosis gene panel (IL17RA, EMC1, PLD3, GDF15, FKBP10, ADAM9, and PLAT) highlight significant pathways related to sarcoidosis pathogenesis. An increased expression of interleukin 17A (IL-17A) has been described in patients with sarcoidosis, and IL-17A-positive CD4+ T cells have also been shown to infiltrate sarcoid lung lesions from patients at different stages of disease evolution [80,81]. Consequently, IL17 receptors’ dysfunction due to either mutations or viral targeting may have significant effects on various functions of IL-17A in the host’s defense against various microbial pathogens and tissue inflammation. Notably, JAK/STAT signaling may have an essential mediating role by transducing signals from IL-17 receptors, and STAT1 expression was found to increase in human sarcoid tissues relative to controls [82]. Another F-SARCO-CoV2 interactome gene identified in our study is PLAT (Plasminogen Activator Tissue type), which encodes a secreted serine protease that converts the pro-enzyme plasminogen to plasmin, a fibrinolytic enzyme that interacts with Annexin A2, a receptor for plasminogen at the surface of endothelial cells [83]. Clinically, being affected by sarcoidosis is a risk factor for venous thromboembolism, and an increased rate of thrombosis is now a well-described complication described during SARS-CoV2 infection [84,85].

### 4.5. Endoplasmic Reticulum Stress-Related Pathways

Several host proteins interacting with SARS-CoV2 ORF8 are involved in the early secretory pathway, ER homeostasis and the ER-Golgi intermediate compartment (ERGIC), and the Golgi apparatus homeostasis and functionally, which are the mechanisms involved in ER stress resolution, such as UPR, ER-associated degradation (ERAD), and autophagy. Some of these proteins are encoded by genes mutated in familial sarcoidosis and have interesting functions. The EMC1 gene encodes subunit 1 of the endoplasmic reticulum-membrane protein complex, a highly conserved and ubiquitous multiprotein transmembrane complex, enabling the energy-independent insertion into ER membranes of newly synthesized host and viral membrane proteins [86]. The peroxisomal and mitochondrial function of EMC proteins play a role in sterols, phospholipid metabolism, and biochemical factors involved in sarcoidosis pathogenesis [87,88]. Interestingly, critical factors regulating peroxisomal activity, such as PPAR (Peroxisome Proliferator-Activated Receptor), are down-expressed in alveolar macrophages from sarcoidosis patients, leading to increased local inflammation [89]. Closely associated to ER, phospholipase D3 (encoded by *PLD3*) is expressed into intraluminal vesicles of multivesicular endosomes and colocalized with amyloid precursor (APP). Mutations of PLD3 are considered to be a genetic factor involved in late onset Alzheimer’s disease associated with an increased processing of APP to Aβ [90]. Interestingly, the accumulation of amyloid peptides is a hallmark of sarcoid granulomas [91]. Two genes of the F-SARC-CoV2 and control panels (FKBP10 or FK506-binding protein 10, a molecular chaperone with peptidyl-prolyl isomerase activity, which binds the immunosuppressive drug FK506 or tacrolimus) and ADAM9 (A disintegrin and a metalloprotease 9), interact with cellular factors involved in UPR (unfolded protein response) [92,93]. UPR is a molecular process maintaining cellular homeostasis and having a crucial role in the host’s defense against viral infection. Cellular stresses, such as those induced by viral infection, activate several well-orchestrated processes aimed at either restoring cellular homeostasis or committing cell death. They include the unfolded protein response (UPR), autophagy, hypoxia, ER stress, and mitochondrial function, whose dysregulation may lead to many auto-inflammatory diseases such as sarcoidosis [94]. In the same manner, focus could be placed on EXOSC2 (Exosome Component 2) targeted by the SARS-CoV2 protein NSP8, also known as ribosomal RNA-processing protein-4 (RRP4), an essential component of exosomes or extracellular vesicles acting as intercellular transmitters, and a scavenger of microbial RNA and infectious agents [95]. Broncho-alveolar lavage fluid of sarcoidosis patients showed increased levels of exosomes inducing various pro-inflammatory cytokines, such as IL-1β, IL-6, and TNF (Tumor Necrosis Factor) alpha [96]. One may expect that any deficiency in autophagy—either autophagosome maturation or autolysosome formation—may induce a shift towards the endosomal–exosome pathway through the fusion of autophagosomes to multivesicular bodies (MVB) and the release in exosomes [97,98]. Many reports have clearly established a link between exosome overproduction and endothelial dysfunction, vascular inflammation, coagulation, and thrombosis [99,100]. As shown previously, deregulation of Rac1-related pathways including mTOR appears to be a relevant predisposing factor for familial sarcoidosis [4,25,42]. VPS11 (Vacuolar Protein Sorting 11) and HS2ST1 (Heparan Sulfate 2-O-Sulfotransferase 1), respectively targeted by SARS-CoV2 ORF3A and NSP7 proteins, contribute to early-to-late endosome transition, lysosome biogenesis, and endo-lysosomal trafficking pathways, including autophagy [101,102]. VPS11 is a subunit of the HOPS (HOmotypic fusion and Protein Sorting)/CORVET (Class C Core Vacuole-Endosome Tethering) molecular complex that connects small Rab GTPases to Golgi proteins and autophagosomes, acting in the endosomal fusion process [103]. Rab and Rac1 belong to the Rho GTPase family and act in the regulation of the actin cytoskeleton and many other cellular pathways, such as production of reactive oxygen species and gene transcription [104]. In addition to the implication of dysregulated Rac1-related pathways, genetic variants of some Rab proteins, such as Rab23, correlate with some phenotypes of sarcoidosis [105]. Heparan sulfate proteoglycans (HSPGs), such as HS2ST1, act as signaling co-receptors via interactions of their sulfated glycosaminoglycan chains with numerous signaling molecules, such as Rab and Rac1 Rho GTPases, and play significant roles in cell motility and adhesion to fibronectin and laminin [106]

### 4.6. F-SARCO-CoV Genes Related to Virus Entry

Finally, we would like to focus on two genes (ZDHHC5 and GOLGA7) targeted by SARS-CoV2 SPIKE (S), which mediate receptor binding and cell entry of the virus, and are considered to be the main targets for vaccine and therapeutic development. The S protein plays a key role in the induction of neutralizing-antibody and T cell responses, in addition to protective immunity, during infection with SARS-CoV. Interestingly, ZDHHC5 and GOLGA7 interact to form a catalytically active protein S-acyltransferase complex that is localized to the plasma membrane and likely mediates palmitoylation of the S protein [107,108]. The identification of germline mutations of ZDHHC5 in familial sarcoidosis raises the question of the high susceptibility of such patients to SARS-CoV2 infection and reactivity to anti-S protein antibodies, either natural or vaccine-induced. Similar questions have been raised in several studies for individuals sharing genetic polymorphisms in ACE2 and TMPPRSS2, respectively, as the receptor and catalytic co-receptor of SARS-CoV2 [109].

### 4.7. Impact of Genetic Data on Sarcoidosis and SARS-CoV2 Infection

There are several studies concerning SARS-CoV2 infection in patients with sarcoidosis. There is still a controversy concerning whether sarcoidosis patients are at a higher risk of contracting SARS-CoV2 and have a more severe outcome than the general population. Some subgroups of patients with particular sarcoidosis phenotypes or treatments might be at higher risk. However, no study to date has studied the impact of COVID-19 in familial forms of sarcoidosis in conjunction with severe forms of the disease. A large international survey based on a questionnaire suggested that the incidence of SARS-CoV2 symptomatic infection does not increase in sarcoidosis when all clinical forms of this disease are considered [8]. Nevertheless, the clinical impact of COVID-19 appears to be more frequent in sarcoidosis patients having a severe pulmonary or neurological involvement [110]. Some sarcoidosis-induced phenotypes are clearly associated with an increased risk of severe outcome and/or death, such as severe basal impaired pulmonary function in three independent studies [111,112,113], and chronic liver and kidney disease [114,115], together with classical unfavorable factors well known in the general population, such as diabetes and obesity. Interestingly, Afro-American patients with chronic sarcoidosis treated with anti-rheumatic or anti-TNF (Tumor Necrosis Factor) therapies do not appear to be at higher risk of life-threatening complications after SARS-CoV2 infection [116]. A similar conclusion concerns patients treated by prednisone at a dose higher than 10 mg per day, whereas Rituximab, a chimeric anti-CD20 antibody used in several auto-inflammatory diseases, appears to increase the impact of COVID-19 disease [8]. These observations suggest the importance of respecting a balance of treatments for sarcoidosis in a pandemic situation, in order to avoid worsening pulmonary function, which in itself will be highly deleterious in a patient infected with the virus. In our study, we have no information about the occurrence of COVID-19 in our familial cases of sarcoidosis and control individuals. Nevertheless, we may suggest that retrospective and prospective studies will be needed in a subset of (familial and sporadic) sarcoidosis patients that have been genotyped, to evaluate the real impact of viral infection in patients sharing pathogenic mutations in genes critically involved in the autophagy/mitophagy processes, and the immune pathways involved in the response to coronavirus infections.

### 4.8. Pharmacological Impact of Identified Pathways

As discussed by Badary in a recent review on pharmacogenomics and COVID-19, the search for common genetic pathways in pathological conditions such as sarcoidosis and the SARS-CoV2 host-cell interactome may be essential for the characterization of therapeutic repositioning [117]. Our data highlight the fact that mitochondrial dysfunction, both at the metabolic and mitophagy levels, is a crucial step in antiviral defense. Drugs’ interactions within mitochondria may target different cytochromes and have functional consequences by activating mitophagy and autophagy, as already observed with corticosteroids, which are recognized to significantly improve the prognosis of both SARS-CoV2-induced pulmonary lesions and the chronic course of sarcoidosis [118,119]. Interestingly, dexamethasone induces Heme oxygenase 1 (HO-1 encoded by HMOX) expression, a primary antioxidant enzyme involved in heme group degradation [120,121]. HO-1 has a major anti-inflammatory role and the therapeutic modulation of this enzyme has been evaluated in a therapeutic strategy in COVID-19 treatment [122]. Natural compounds, such as vitamin D, resveratrol, or curcumin, activate mitophagy/autophagy and could be putative therapeutic adjuvants in COVID-19 [123,124,125]. As discussed previously, the use of Janus kinase (JAK) inhibitors, such as Tofacitinib, was recently reported in sarcoidosis patients [126]. During viral infections, the IFN/JAK/signal transducer and activator of the transcription (STAT) signaling pathway activates TRIM21 (Tripartite Motif Containing 21), a cytosolic ubiquitin ligase, which catalyzes the polyubiquitination of MAVS, a crucial biochemical step to promote TBK1 recruitment to MAVS [127]. Interestingly, TRIM21 appears to be a key player in preventing efficient adenovirus-based gene delivery and vaccination [128]. Many pro-inflammatory factors involved in cytokine storms during COVID-19 are antagonized by JAK inhibitors, such as IL-6, a multifunctional cytokine exerting its action through the IL-6 receptor (IL-6R) and the downstream activation of the JAK/STAT and MAPK (mitogen-activated protein kinase) pathways [129]. Tocilizumab, a humanized monoclonal antibody directly targeting the IL-6R, significantly improves lung function in COVID-19 and was also reported to be efficient in case reports of sarcoidosis-associated posterior uveitis [130,131].

At the crossroads of autophagy and interferon production pathways, our results highlight that TBK1 is a pivotal gene whose mutations may have deleterious effects in both viral-induced diseases, such as COVID-19, and sarcoidosis. Interestingly, the SARS-CoV2 functional interactome described by Hoffmann and collaborators identified MIB1 (Mindbomb 1), an E3 ubiquitin-protein ligase, as a mediator of TBK1 activation and, consequently, having a role for integrating signals in response to viral infection through RIG-like receptors and IRF3 [10]. TBK1 is one of the major genes involved in predisposition to Amyotrophic Lateral sclerosis and frontotemporal dementia, and one may expect that therapeutic modulation of TBK1-related pathways will be a future challenge in such indications and, more specifically, in viral infections and sarcoidosis [132,133,134].

Another interesting compound is quercetin, a naturally derived PDE4 (Phosphodiesterase 4D)-selective inhibitor found in fruits, vegetables, and tea, with antioxidant and anti-inflammatory properties, which has been proposed in the treatment of COVID-19 [135]. By regulating cAMP levels, PDE4D controls mTOR activity through the regulation of RHEB and, consequently, autophagy and mitophagy processes [136]. Other functional targets identified in our genetic screening of sarcoidosis patients highlighted endosomal and lysosomal functions, which are targeted by several drugs, such as chloroquine and hydroxychloroquine. These are two efficient treatments in mild forms of sarcoidosis (moderate hypercalcemia, skin involvement), but whose ineffectiveness in COVID-19 was demonstrated in vivo [137]. The overactivation of IL-17-producing Th17 cells may occur in patients with COVID-19 and the use of monoclonal antibodies against IL-17A (such as secukinumab and Ixekizumab) has been discussed as an efficient treatment to prevent organ injury caused by the immune response to COVID-19 [138]. There are several means to down-regulate the IL-17A downstream effect, including blocking IL-17 itself (by secukinumab and Ixekizumab) or its receptor (by Brodalumab). However, a recent case report suggested a potential causal association of Ixekizumab with the occurrence of cardiac sarcoidosis, suggesting further studies will be needed to clarify whether they could be beneficial for patients [139]. The identification of germline mutations in the gene encoding FKBP10 (FK506-binding protein 10) is interesting due to the fact that it binds FK506 (tacrolimus), an immunosuppressive drug which has been used in cutaneous and cardiac refractory forms of sarcoidosis [140]. Focusing the SARS-CoV2 Spike (S) protein (S) remains a major target in both therapeutics and vaccine strategies against COVID-19. The SARS-CoV2 S protein interacts with the GOLGA7-ZDHHC5 acyl-transferase complex, which mediates palmitoylation on its cytosolic tail, a process that facilitates membrane fusion and is putatively a potential target for therapeutic inhibition [141]. Finally, our work highlights some pathways involved in coagulation and this provides clues to understanding thromboembolic events in both sarcoidosis and COVID-19 [1,142].

### 4.9. Limitations

Our study has several limitations: (1) it is only a descriptive work based on WES genetic screening without any functional studies; (2) the selection of gene variants was performed in the usual manner in WES analysis, i.e., by progressively decreasing the MAF value of variants and using a genotype subtraction protocol with external and, when available, some intrafamilial healthy individuals, a strategy that can be criticized at different levels; (3) the in silico functional evaluation of gene variants remains a complex procedure and needs to cross various databases with putative contradictory results; and (4) mutation databases and protein network enrichment software use published and hopefully confirmed results, and may therefore be incomplete regarding genes’ functions, reflecting the fact that their interactions are poorly understood. We considered that a descriptive and literature-based approach could be more relevant than a statistical one. As suggested recently by Perfetto and collaborators, gene set enrichment methods frequently exploit molecular interaction networks and miss functional consequences—i.e., activation or repression of intracellular pathways—of these interactions. [143].

**Figure 6:**
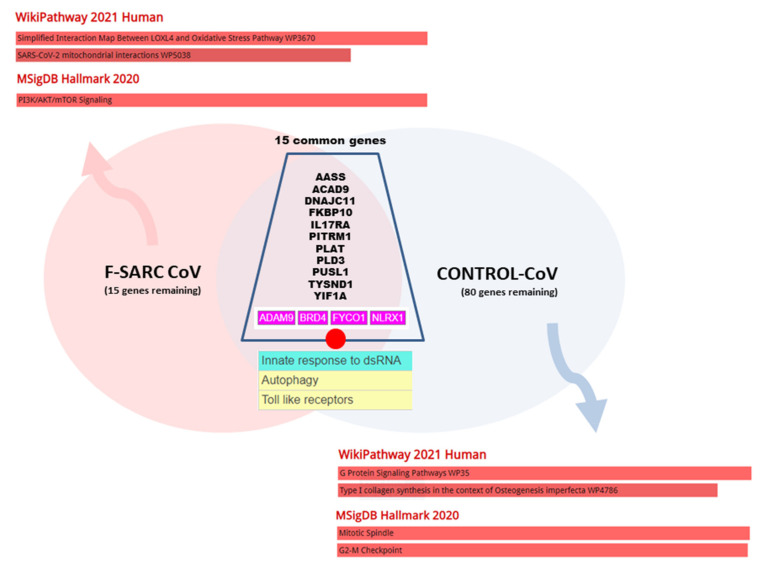
Venn diagram of the clusters of gene sharing pathogenic variants in F-SARC-CoV (sarcoidosis) and control series. Gene acronym descriptions are in the text.

As shown in Figure 6, an online resource was used to compute the functional modules linked to the SARS-CoV2-host interactome and the cellular pathways (SIGNOR 2.0 - The Signaling Network Open Resource—Available online: https://signor.uniroma2.it/covid/, accessed on 24 May 2021), and thus assess the differences in the pathological impact of gene mutations between the F-SARC-CoV and the control panels. For this purpose, we identified 15 genes of the SARS-CoV2 GHI common to the sarcoidosis-related and control series. Two web-based network enrichment servers (WIKIPATHWAYS 2021 and MsigDB 2020) were used to analyze the remaining genes (15 for F-SARC-CoV and 80 for Control-CoV) through ENRICH.R, and identified different top-enriched pathways in sarcoidosis (oxidative stress, *p* = 0.0000192, and SARS-CoV2 mitochondrial interactions, *p* = 0.003122, respectively) and control individuals (G protein signaling, *p* = 0.007176, and mitotic spindle, *p* = 0.008351, respectively).

## 5. Conclusions and Perspectives

Our main concern in this work was the search for genetic factors that likely increase the predisposition of sarcoidosis patients with severe forms of SARS-CoV2 infection. For this purpose, we analyzed gene pathogenic variants observed in 13 families predisposed to sarcoidosis and compared the data to the genes belonging to the SARS-CoV2 host-cell protein-protein interactome. In addition, we performed the same study in a series of healthy control subjects. Our results showed that both populations (sarcoidosis and controls) shared pathogenic mutations in around 10% of the genes listed in the SARS-CoV2 interactome and putatively disrupting interactions between host and viral proteins during infection. The main pathway affected is the RIG-I/MDA-5 pathway, which subsequently attenuates antiviral immunity and enhances viral replication. Sarcoidosis patients also accumulate a significant number of mutations in genes related to intracellular trafficking, and autophagy and mitophagy regulation, with a remarkable focus on the mTOR functional hub. Consistent with the data published by the “COVID Human Genetic Effort”, we observed that the TBK1-related gatekeeper pathway, both in autophagy and interferon production, is probably a critical functional node whose maintenance is fundamental in antiviral defense [45]. This raises the question of the susceptibility of sarcoidosis patients to SARS-CoV2 infection, and the possibility that vaccine procedures’ side effects in individuals may share germline defects in these pathways. Finally, we would like to hypothesize that sarcoid granulomas may be the pathogenic expression of a common reaction to various environmental triggers and viral infection, in which symptomatic forms leading to sarcoidosis are only the tip of the iceberg.

## Figures and Tables

**Table 1:** The list of genes identified in familial sarcoidosis (F-SARC) and control individuals (CTRL) and belonging to the SARS-CoV2 GHI (Gordon and Hoffmann interactome) [9,10]. Lines highlighted in pink indicate the genes found in both the F-SARC-CoV and the F-CTRL control series 3.3. Gene set enrichment of genes identified in familial sarcoidosis and controls.

Gene	Denomination	SARS-CoV2	Functional Key Words	Identified in
Symbol				
	**Virus—Host cell interacting proteins involved in autophagy, mitophagy and mitochondrial processes**			
**YIF1A**	Yip1 interacting factor homolog A	**M**	Mitochondrial factor involved in autophagy	F-SARC + Control
**PITRM1**	Pitrilysin metallopeptidase 1	**M**	Member of the mitochondrial OXPHOS system	F-SARC + Control
**FASTKD5**	FAST kinase domain 5	**M**	Regulation of all mitochondrial-encoded RNA	F-SARC
**STOM**	Stomatin	**M**	Cellular/mitochondrial ion channel activity	F-SARC
**AASS**	Aminoadipate-Semialdehyde Synthase	**M**	Saccharopine-induced mitochondrial damage	F-SARC + Control
**TBK1**	TANK Binding Kinase 1	**Nsp13**	Regulation of IFN-I and mTOR, aggregates with MAVS	F-SARC
**GOLGA2**	Golgin subfamily A member 2	**Nsp13**	Golgi-derived vesicles traffic regulation, endosomes	F-SARC
**GOLGA3**	Golgin subfamily A member 3	**Nsp13**	Golgi-derived vesicles traffic regulation, endosomes	F-SARC
**GOLGA7**	Golgin subfamily A member 7	**SPIKE**	Interacts with ZDHHC5, Golgi vesicles traffic regulation	F-SARC
**GOLGB1**	Golgin subfamily B member 1	**Nsp13**	Golgi-derived vesicles traffic regulation, endosomes	F-SARC
**TYSDN1**	Trypsin Like Peroxisomal Matrix Peptidase 1	**Nsp12**	Component of peroxisomes, role in pexophagy	F-SARC + Control
**ECSIT**	ECSIT Signaling Integrator	**Orf9b**	Mitochondrial factor, regulation of viral signaling	F-SARC
**NLRX1**	**NLR Family Member X1**	**Orf9b**	Mitochondrial factor, interfere in MAVS-RIG1 signaling	F-SARC + Control
**ACAD9**	**Acyl-CoA Dehydrogenase Family Member 9**	**Orf9b**	Component of the mitochondrial complex I	F-SARC + Control
**PUSL1**	tRNA pseudouridine synthase-like 1	**Orf8**	Mitochondrial protein for efficient mt-RNA translation	F-SARC + Control
**DNAJC11**	DnaJ homolog subfamily C member 11	**Nsp4**	Component of the mitochondrial complex I	F-SARC + Control
**BRD4**	Bromodomain-containing protein 4	**E**	Regulation of autophagy, mitophagy through PINK1	F-SARC + Control
	**Virus—Host cell interacting proteins involved in Rho-GTPases (Rab/Rac1) related pathways**			
**VPS11**	Vacuolar protein sorting-associated protein 11 homolog	**Orf3A**	Connects Rab GTPases to Golgi and autophagosome	F-SARC
**PDE4DIP**	Phosphodiesterase 4D Interacting Protein	**Nsp13**	PDE4D-related regulatory role in Golgi microtubules	F-SARC
**FYCO1**	FYVE And Coiled-Coil Domain Autophagy Adaptor 1	**Nsp13**	Rab7-mediated transport of late endosomes	F-SARC + Control
**HS2ST1**	Heparan Sulfate 2-O-Sulfotransferase 1	**HS2ST1**	Involved in Rab and Rac1 signaling	F-SARC
	**Virus-host cell interacting proteins involved in ER-associated vesicular traffic, extracellular matrix, and coagulation**			
**ZDHHC5**	Zinc finger DHHC-type palmitoyltransferase 5	**SPIKE**	Intracellular protein trafficking and S-acylation	F-SARC
**EMC1**	ER Membrane Protein Complex Subunit 1	**Orf8**	The largest component of ER transmembrane complex	F-SARC
**PLD3**	Phospholipase D3	**Orf8**	ER transmembrane protein, acts on amyloid secretion	F-SARC + Control
**GDF15**	**Growth Differentiation Factor 15**	**Orf8**	Role in IPF, correlation with pulmonary function	F-SARC
**FKBP10**	FK506-binding protein 10	**Orf8**	Involved in transmembrane signal transducers IRE1	F-SARC + Control
**ADAM9**	Disintegrin metalloproteinase domain-containing protein 9.	**Orf8**	Involved in transmembrane signal transducers IRE1	F-SARC + Control
**IL17RA**	Interleukin 17 Receptor A	**Orf8**	Innate immune signaling, role in thrombosis	F-SARC + Control
**PLAT**	Plasminogen Activator, Tissue Type	**Orf8**	Interaction with annexin A2, role in coagulation	F-SARC + Control
**EXOSC2**	Exosome Component 2	**Nsp8**	Extracellular vesicles, clearance of viral particles	F-SARC
**UGGT2**	UDP-Glucose Glycoprotein Glucosyltransferase 2	**Orf8**	Provides quality control for protein folding in the ER	CTRL

## Data Availability

Data available on request.

## Supplementary Materials

The following are available online at https://www.mdpi.com/article/10.3390/cells10081995/s1, Table S1: Description of gene variants found in patients of 13 sarcoidosis families. Table S2: The list and brief description of genes sharing rare (MAF < 0.05) and pathogenic mutations in the control series. Table S3: Intragenic variants of genes identified in familial sarcoidosis and belonging to the SARS-CoV2 host-pathogen Gordon Hoffmann interactome (GHI) (the F-SARC-CoV panel).
