# Supplementary material for: Autophagy and Mitophagy-Related Pathways at the Crossroads of Genetic Pathways Involved in Familial Sarcoidosis and Host-Pathogen Interactions Induced by Coronaviruses
Source: Cells. 2021 Aug 5;10(8):1995. doi: 10.3390/cells10081995 (PMC8393644; doi:10.3390/cells10081995)
Supplement: Supplementary file 1 [file cells-10-01995-s001.zip › Tables Descriptions.pdf]

Table S1. Description of gene variants found in all patients of the 13 sarcoidosis families (F1 to F13). For a single family, the variants shown are those which are been identified in all the intrafamilial affected cases. For families F1, F2, F3, F4, F5 and F13, the availability of intrafamilial healthy controls allowed to perform a genotype subtraction and the variants described here were only found in sarcoidosis confirmed cases (REF). For families F6 to F12, only affected cases have been sequenced and their genotypes have been compared to those of 12 healthy individuals from the Pulmonology department (University Hospital – Lyon – France) and further checked in an in-house database of around 600 whole exome sequencing data. In some cases, a single gene shared variants in two or more families, and only in this case, we have included these variants in Table S1 in order to have an exhaustive evaluation of putative recurrent mutations. From left to right, Table S1 includes the name of the gene (GENE), the international reference of the variants (rs), the EXON number inside the gene, the CODON position, the type of variant, the effect on protein structure, the in silico pathogenic evaluation by SIFT (0 to 1 from pathogenic to benign, respectively) and POLYPHENV2 (0 to 1 from benign to pathogenic respectively), two parameters described by Mutation Taster software and respectively the conservation in various species (0 to 1 from not conserved to highly conserved, respectively) and the Grantham score (from 5 – low to 215 – high to predict the physicochemical distance between two amino acids), the transcript accession number (NM), the families reference (F), the minor allele frequency (MAF), the CADD (Combined Annotation-Dependent Depletion) score summarizing the criteria of pathogenicity and clinical significance of a variant (from low < 20 to middle 20-25 and high > 25), the position of nucleotide change in cDNA, the chromosomal / genomic position and lastly the mean WES depth of readings of the variants. Some of the variants have putative effects on splicing and this was indicated as SPLICING, both at the DONOR (exon-intron or ACCEPTOR (intron-exon) sides. All the date were checked by Alamut® Visual software which integrates missense variant pathogenicity prediction tools and in silico study of variants' effect on RNA splicing, allowing the assessment of their potential impact on splice junctions and splicing regulatory sequences. Alamut® Visual helped us also to exclude well-known mutations identified in recessive diseases for those genes and related to various known genetic diseases.

Table S2. Genes of the control series crossing with the SARS-CoV2 GHI interactome. The acronyms of the 95 genes of the Control-SARS-CoV2 GHI are detailed and those common with the F-SARC-CoV GHI highlighted in yellow (column B). A brief description of the major function is provided in column C.

Table S3. Intragenic variants of genes identified in familial sarcoidosis and belonging to the SARS-CoV2 host-pathogen GORDON-HOFFMANN interactome (the F-SARC-CoV panel). Abbreviations: rs accession number to refer to specific Single Nucleotide variants; MS: missense, FS: frameshift, IF-DEL: in frame deletion variants; NM: NCBI reference sequence of mRNA; CADD: Combined Annotation Dependent Depletion, a tool for scoring the deleteriousness (> 20) of single nucleotide variants as well as insertion/deletions variants in the human genome; POLYPHEN: the evaluation of in silico pathogenicity by the Polyphenv2 web server (0: not pathogen, 1: highly pathogen); M/Taster: scoring the variants by the Mutation Taster software, (ex: 1-160), the left number indicate the degree of conservation in species (1= highly conserved, 0 not conserved), the right number is the Grantham score indication the physicochemical impact of the amino acid substitution (0: no impact - > 100 : high impact); MAF: minor allele frequency; DEPTH: indicate the number of reads during the next generation sequencing process; nd: not defined.
